# Fairness Considerations When I Know More than You Do: Developmental Comparisons

**DOI:** 10.3389/fpsyg.2012.00424

**Published:** 2012-10-19

**Authors:** Sandy Overgaauw, Berna Güroğlu, Eveline A. Crone

**Affiliations:** ^1^Institute of Psychology, Leiden UniversityLeiden, Netherlands; ^2^Leiden Institute for Brain and CognitionLeiden, Netherlands; ^3^Department of Psychology, University of AmsterdamAmsterdam, Netherlands

**Keywords:** development, empathic concern, fairness, perspective taking, prosocial behavior, social decision making

## Abstract

The Ultimatum Game (UG) is a valuable paradigm to study fairness considerations. Here, we tested developmental differences between altruistic and strategic motivations in fairness considerations using a version of the UG with hidden conditions. Participants were proposers and could divide coins between themselves and an anonymous other. Hidden information conditions involved division of coins where some coins were only visible to the participant (e.g., 8/2 condition where, from the total of 10 coins, 8 coins were visible to both players and 2 coins only visible to the proposer). In total, 22 young adults and 79 children between ages 8 and 13 played multiple one-shot versions of the UG with hidden conditions with anonymous others. Overall analyses confirmed validity of the task and showed that participants of all age groups had strategic intentions. Specific task analyses revealed that adults divided the coins equally in the standard UG conditions, but gave less to the second player in the hidden information conditions. The developmental comparisons revealed an age × condition interaction, such that adults and 10- to 12-year-old children differentiated between standard and hidden conditions more than 8- to 9-year-old children. These findings indicate that young children have a basic understanding of different strategic motives, but that behavior of adults and older children is driven more by strategic intentions.

## Introduction

Making fairness related decisions is a common and important component of social interactions. These decisions are based on different underlying motives, including the relevance of true fairness and the need to comply with generally applicable social norms (Rilling and Sanfey, 2011). Experimental tasks based on economic exchanges have proven successful in examining people’s strategies in bargaining situations and the factors that influence their decision-making. One of these tasks is the well-known Ultimatum Game (UG). In this two-player bargaining game, the first player (i.e., the proposer) makes an offer to divide the stake, for example 10 coins, between the two players. The second player (i.e., the responder) decides either to accept or reject the proposed division. If the offer is accepted, the coins are divided according to the offer of the proposer. In case the responder rejects the proposed offer, both parties receive nothing (Güth et al., 1982).

Many studies have examined what motivates proposers to offer an equal distribution instead of a “game theoretic” smallest possible offer in the UG context (Fehr and Schmidt, 2006). Contrary to what might be expected, people are not always primarily motivated to maximize their own outcome and seem to care for equality. A commonly given explanation is that proposers are motivated by considerations of fairness (Binmore et al., 1991; Pillutla et al., 1996; Fehr and Schmidt, 2006; Reuben and van Winden, 2008, 2010), which might be partially caused by the acquired social norms of our society (Sally and Hill, 2006; Tomasello and Vaish, in press).

However, besides the “altruistic” willingness to be fair (Kagel et al., 1995; van Dijk and Vermunt, 2000; Kohler, 2011), players might also be motivated by the fear of rejection (van Dijk et al., 2004), resulting in strategic action (Güth et al., 1982; Binmore et al., 1985). That is, if the offer is beneficial for the proposer but not for the responder (i.e., an unfair split), the responder may reject the offer. In this case, the responder prefers that both parties receive nothing over an unfair split. Thus, the proposer in the UG may wish to maximize self-profit, but may take the perspective of the responder who might reject an unfair offer, resulting in the socially desirable fair offer. In this case, fairness requires the ability to take the perspective of others into account (Güroğlu et al., 2009; Steinbeis et al., 2012). In short, fairness considerations might be based on two different processes: a true belief in fairness (i.e., an altruistic motivation), and a fear for rejection (i.e., a strategic motivation).

### Developmental comparisons

Several studies have shown that children under the age of 7 prefer distributions in their own favor and that preference for fair distributions increases with age (Benenson et al., 2007; Fehr et al., 2008; Gummerum et al., 2008; Blake and Rand, 2010; Blake and McAuliffe, 2011). These developmental differences between ages 3–4 and 8 years have been interpreted as an increased preference for equity (Fehr et al., 2008; Tomasello and Vaish, in press). For example, Blake and McAuliffe (2011) examined inequity aversion among 4- to 8-year-olds, where participants could use an apparatus that divided candy by pulling a green or a red handle, which allowed them to either accept or reject the offer. The game consisted of two conditions: disadvantageous inequity (1/4) and advantageous inequity (4/1). In both conditions the alternative distribution was the equity distribution (1/1), controlling for a default rejection tendency. Interestingly, only the 8-year-olds showed a preference for fairness in both advantageous and disadvantageous inequity conditions, willing to sacrifice their own coins in order to provide equity in the advantageous inequity condition.

This developing preference for fairness over self-interest across childhood is thought to partly depend on the acquisition of perspective taking (PT) abilities, which enables children to take another person’s view (Harbaugh et al., 2003; Takagishi et al., 2010). This progressing ability to take the perspective of others, which is defined here as the ability to understand thoughts and intentions by others and willingness to act on this understanding, can subsequently result in the development of strategic behavior. One way of examining strategic intentions behind fairness considerations is comparing offers made by proposers in the UG and the Dictator Game (DG). The DG differs from the UG in the way that proposals in the DG cannot be rejected by the receiver; the proposed offer determines the outcome for both players. If fairness is the driving force behind offering equal distributions, the proposals should be the same in both experimental games. Indeed, prior studies have demonstrated that fair proposals increase with age in the UG relative to the DG, with the greatest change occurring between ages 7 and 10 years (Harbaugh et al., 2003; Leman et al., 2008; see also Steinbeis et al., 2012, who replicated the same finding in children between ages 6 and 13 years).

Güroğlu et al. (2009) studied proposer behavior in 9–18 year olds using an adapted version of the DG and the UG, which was developed to study the role of PT. This experimental variant, also referred to as the mini-UG, gave proposers two options for distributing 10 coins. One offer was always an unfair distribution (8 coins for proposer, 2 for responder), and this offer was presented next to another distribution which could be: 2 coins for the proposer and 8 for the responder (hyper-fair condition), 5 coins for both (fair condition), or 10 coins for the proposer and none for the responder (hyper-unfair condition). The findings showed that with increasing age, participants were increasingly strategic in the offers they made, such that they differentiated more between the three conditions based on the alternative distribution that was presented.

Taken together, prior developmental studies show evidence for an increase in offers in the UG between ages 7–8 and 12–13 years, suggesting a developmental change in fairness considerations in this period. The comparison between UG and DG behavior suggests that even young children are able to act strategically (Harbaugh et al., 2003), but that these strategic intentions increase with age (Steinbeis et al., 2012). This leads to the question whether the developmental changes in UG offers can indeed be attributed to strategic (i.e., higher offers out of fear for rejection) rather than altruistic (i.e., higher offers because of equity/fairness preference) motivations behind fairness considerations.

### Dissociating altruistic versus strategic considerations

Several researchers have employed a modified version of the UG with hidden information conditions in order to dissociate between altruistic and strategic considerations in UG offers (Kagel et al., 1995; van Dijk et al., 2004; van Beest et al., 2011). Hidden information refers to information that is only available to the proposer and that can be used in his/her own benefit. For example, it can be the case that the proposer has 10 coins to share with the responder, but the responder thinks that there are only 8 coins in the game (i.e., 8/2 condition where from the total of 10 coins, 8 coins were visible to both players and 2 coins only visible to the proposer). If the proposer’s decision was based on altruistic motivations, he or she would offer 5 coins (a true fair split). However, if the proposer is motivated by strategic considerations, he or she would offer 4 coins to the responder, knowing that the responder will *think* this is a fair split. These hidden information conditions give the proposer the opportunity to act in an *altruistically* fair manner or in a *seemingly* fair manner, by making a strategic offer and using the hidden information to maximize self-gain (Kagel et al., 1995; van Dijk et al., 2004; Koning et al., 2011).

Prior studies demonstrate the influence of various sorts of hidden information on proposer behavior in adults. Overall, it was found that proposers make more equal offers in complete information conditions than in the hidden information conditions and take advantage of hidden information by making seemingly fair offers and maximizing self-gain (Camerer and Loewenstein, 1993; Straub and Murnighan, 1995; Rapoport and Sundali, 1996; Murnighan and Saxon, 1998; van Beest et al., 2011). The UG with hidden information therefore proves valuable to answer questions related to the development of fairness considerations based on altruistic versus strategic motivations.

### The current study

In the present study, we asked children between ages 8–13 and adults to play the role of the proposer in the hidden information version of the UG. Our main expectation was to find an increase in strategic fairness with age. Specifically, we expected adults to make higher offers than children in the complete information trials compared to the hidden information trials, based on the assumption that adults are better able to take the perspective of others and make strategic offers based on what they are willing to receive themselves (Page and Nowak, 2002). This pattern of behavior was expected to emerge between ages 8 and 13 (Harbaugh et al., 2003; Blake and McAuliffe, 2011; Steinbeis et al., 2012). We also aimed to investigate the role of PT skills in fairness considerations across adolescence using a self-report index of PT abilities.

## Materials and Methods

### Participants

In total there were 101 participants, including 22 adults between ages 18 and 25 years (mean age = 20.23, SD = 1.23, 5 males and 17 females) and 79 children between ages 8 and 13 years. The children were divided into four age groups: 8- to 9-year-olds (mean age = 8.5 years, SD = 0.27, 4 boys and 10 girls), 10-year-olds (mean age = 10, SD = 0.71, 7 boys and 11 girls), 11-year-olds (mean age = 11.41 SD = 0.28, 14 boys and 13 girls), and 12-year-olds and older (mean age = 12.37, SD = 0.32, 13 boys and 7 girls; see Table 1). Of this sample, eight participants were excluded because of missing data and eight because they were extreme outliers (e.g., only pressing button “1” for the whole task). All children were recruited from elementary schools and participated voluntarily with the consent of the school and their parents. Adults were contacted through a university recruitment program and were tested in a quiet laboratory, comparable to the test session of the children.

**Table 1:** **Descriptives of sample sizes, gender distribution, and mean age in years (SD) of the five age groups**.

	Group				
	1	2	3	4	5
Number of participants	14	18	27	20	22
Female	71%	61%	48%	35%	77%
Mean age	8.5	10	11.41	12.32	20.23
SD	0.27	0.71	0.28	0.32	1.23

### The hidden ultimatum game

The computer task was a variant of the classical UG. Written instructions were presented on the computer screen. Participants were told that they had to divide a certain number of coins, each with a value of 1 Euro, between themselves and the anonymous second players (i.e., the responders); the participants were told that the game was played online through an Internet connection with the responders. They were also told that the responder could accept or reject the offer they made: if the responder rejected the offer, both players would receive nothing; if the offer was accepted, the coins would be distributed as suggested by the participant. Subsequently, they were informed about the existence of two kinds of stakes: stakes with complete information and stakes with hidden information. In case of the complete information, both players were aware of the amount of coins in the game. In case of hidden information, only the proposer knew the total amount of coins that could be shared, and the responder was not aware that there was hidden information.

The task consisted of 15 conditions with three levels for the total number of coins (8, 9, or 10 coins in the game) and five levels for the number of hidden coins (0, 2, 3, 4, or 5 hidden coins). Each condition was offered twice in a random distribution yielding a total number of 30 trials.

**Figure 1:**
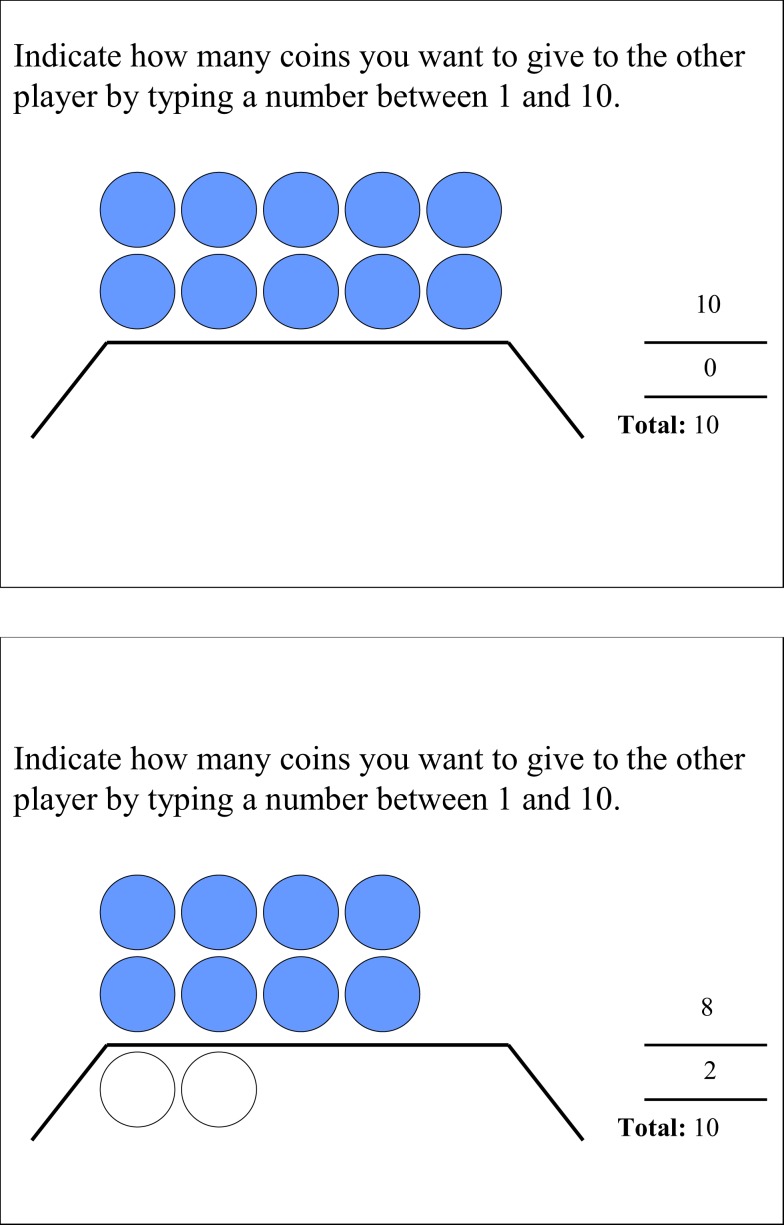
**Complete and incomplete information conditions visible to the participant in the role of proposer**. The blue coins indicate visibility to both proposer and responder. The white coins indicate a hidden condition and are only visible to the proposer.

Each trial started with a fixation cross with duration of 1 s, followed by the stimulus, which was response terminated. Hidden information trials involved blue coins visible to both the proposer and the responder, and white coins only visible to the proposer (see Figure 1). When the stimulus appeared, the proposer could make an offer from 0 to a maximum of 8, 9, or 10 coins (depending on the condition) by pressing an associated key on the keyboard. After the proposer made the offer, a screen appeared for 3 s, which signaled that the receiver was making his/her decision. The decision of the receiver was not presented to the proposer to avoid learning effects. Following the decision-time screen, a feedback screen of 5 s was presented which summed up the offer and indicated what the proposer would retain if the offer would be accepted.

### The interpersonal reactivity index

In order to measure the cognitive component of empathy, participants filled out the PT subscale of the Interpersonal Reactivity Index (IRI; Davis, 1980). This self-report questionnaire has been used extensively with children and adolescents before (Litvack-Miller et al., 1997; Eisenberg et al., 1999; Gleason et al., 2009). The PT subscale explicitly measures the cognitive tendency to see things from the point of view of others, without necessarily experiencing any affective response. The subscale consists of six items to be answered on a five point Likert scale with (1) completely untrue, (3) in between, and (5) completely true. Cronbach’s alpha for the PT subscale was 0.71.

### Procedures

All participants were tested in a quiet laboratory or classroom. They were told that they were going to play an online game with anonymous same-age others from a different school or university. In reality, the other person was not present and waiting time was computer-simulated. It was explained that the computer would randomly choose trials at the end of the experiment, which would be paid to the participants as well as to the second players. It was thus emphasized that their final reward depended on their choices during the game *and* the acceptance of the offers by the responder.

To make sure that the youngest participants also understood the instructions, the experimenters read the instructions out loud and used chocolate coins to explain the concept. They were explained that there were no right or wrong answers and that they could rely on their own judgment. After the instructions, the task was completed individually on a laptop computer. The task started with eight practice trials, followed by 30 experimental trials. The task was self-paced and all participants completed the game within 15 min. Following the task, the participants filled out the IRI questionnaire (Davis, 1980).

The children were told beforehand that they could choose a small present from the money they would earn in the game. All of the presents the children could choose from had approximately the same value. Adults were told that they would receive the money they had won during the game. All adults were paid a fixed amount of 6.50 Euros (approximately 8 dollars). Neither children nor adults indicated to have any doubts about the genuineness of the task and/or the outcome of their payments.

## Results

### Preliminary analyses IRI

In order to examine age differences in PT skills on the self-report scale of the IRI, we first performed a univariate analysis of variance (ANOVA) with PT as the dependent variable and age group as a fixed factor. Results showed a main effect of age [*F*(4, 89) = 3.65, *p* < 0.01], which indicates that the age groups differed on their PT skills. Tukey *post hoc* tests demonstrated a difference between the youngest and the oldest age group, where 20-year-olds scored significantly higher than the 8/9-year-olds (*M* = 15.95, SD = 2.73 and *M* = 10.75, SD = 4.79, respectively). The remaining age groups did not differ from each other (10-year-olds: *M* = 14.06, SD = 6.47, 11-year-olds: *M* = 13.4, SD = 3.14, 12-year-olds: *M* = 12.47, SD = 3). The correlation between age and PT skills was also significant (*r* = 0.29, *p* < 0.01).

### Preliminary analyses hidden UG

**Figure 2:**
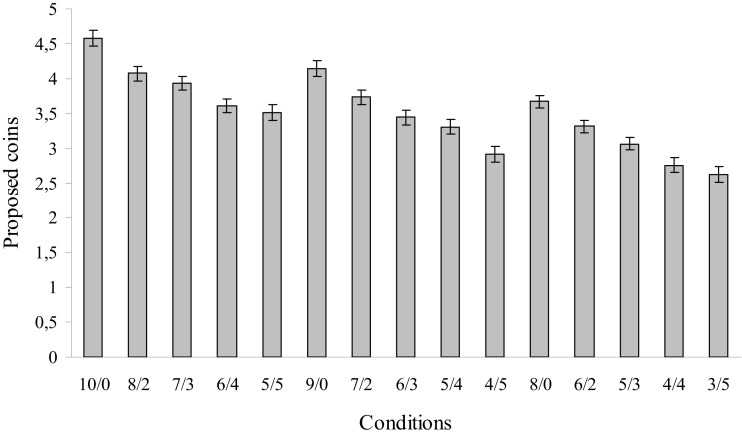
**Mean offers and standard errors of offers made in the three complete information conditions (10/0, 9/0, and 8/0) and the 12 hidden information conditions with two (8/2, 7/2, and 6/2), three (7/3, 6/3, and 5/3), four (6/4, 5/4, and 4/4), and five hidden coins (5/5, 4/5, and 3/5)**.

First, we tested whether the context of the 15 conditions resulted in different offers. A 3 (total coins) × 5 (hidden coins) repeated measure ANOVA across participants indeed resulted in a main effect of total number of coins [*F*(1, 88) = 103.29, *p* < 0.01], as well as a main effect of the number of hidden coins [*F*(1, 88) = 425.72, *p* < 0.01]; there was no significant interaction. First, as can be seen in Figure 2, participants offered a higher amount of coins when there were more coins to be divided. Second, participants gave fewer coins when there was more hidden information. Thus, the main effects validated that the task was successful in measuring fairness considerations under hidden conditions.

The interaction between 3 (total coins) × 5 (hidden coins) × 5 (age group) was not significant. One reason for not finding age effects in this general analysis could be the large number of conditions, which undermined smaller age effects in strategic intentions. The unequal trials (counting up to an uneven number of total coins) were added for exploratory reasons, in order to see how participants would act in reaction to trials in which the participants are forced to choose an unequal distribution. A limitation of these trials is that it is not possible to distinguish between altruistic and strategic trials when the total number of trials is unequal (in essence, on these trials). For this reason, we performed additional tests on the equally numbered trials and as can be seen in the subsequent analyses, when we tested for specific comparisons for fairness considerations, the expected age effects emerged. Next, we present how these considerations are present in different age groups.

### Age comparisons

In our analyses examining age effects, we only included the offers that contained an even number of coins (total 8 or total 10), and an even number of hidden coins (0, 2, or 4). These trials were selected because they allow for direct comparisons of one factor while keeping other factors stable (e.g., 8/0 versus 8/2 tests for the role of hidden information while keeping total number of coins stable). Thus, the analyses below focus on specific questions targeted in this experiment. See Table 2 for an overview of mean offers per condition and age group.

**Table 2:** **Descriptives of mean offers of the 15 conditions in five age groups**.

	Mean age				
	9 Years	10 Years	11 Years	12 Years	20 Years
	*M* (SD)	*M* (SD)	*M* (SD)	*M* (SD)	*M* (SD)
**TOTAL OF 10 COINS**					
10/0	4.17 (1.71)	4.47 (1.05)	4.70 (0.75)	4.50 (1.1)	4.88 (0.61)
8/2	3.38 (1.46)	4.08 (1.23)	4.04 (1.03)	4.12 (0.93)	4.33 (0.48)
7/3	3.83 (1.27)	3.83 (1.06)	3.70 (0.84)	4.15 (1.06)	4.10 (0.54)
6/4	3.25 (1.12)	3.83 (1)	3.70 (0.84)	3.56 (1.09)	3.64 (0.73)
5/5	3.33 (1.8)	3.86 (1.26)	3.36 (0.9)	3.32 (1.26)	3.43 (0.75)
**TOTAL OF 9 COINS**					
9/0	3.79 (2.15)	4.28 (1.27)	3.88 (0.99)	4.12 (0.96)	4.38 (0.59)
7/2	3.29 (1.42)	3.75 (1.44)	3.68 (1.04)	4.03 (0.86)	3.88 (0.31)
6/3	3.25 (1.79)	3.53 (1.27)	3.22 (0.82)	3.71 (1.03)	3.38 (0.5)
5/4	3.54 (1.5)	3.61 (1.36)	3.32 (0.78)	3.03 (1.14)	3.14 (0.48)
4/5	2.79 (1.66)	3.14 (1.29)	2.94 (1)	2.97 (1.15)	2.71 (0.9)
**TOTAL OF 8 COINS**					
8/0	3.08 (1.08)	3.81 (0.88)	3.60 (0.79)	3.65 (0.77)	4.10 (0.54)
6/2	3.21 (1.36)	3.31 (0.96)	3.22 (0.75)	3.50 (0.9)	3.38 (0.5)
5/3	2.71 (0.94)	3.33 (1.28)	2.94 (0.75)	3.24 (1.05)	3.00 (0.55)
4/4	2.71 (1.34)	2.94 (1.38)	2.84 (0.66)	2.68 (1.2)	2.60 (0.74)
3/5	2.50 (1.9)	2.86 (0.97)	2.54 (0.95)	2.59 (1.18)	2.52 (0.75)

The first question concerned whether there were age differences in the *no-hidden information condition*. A repeated measure analysis (ANOVA) with amount of coins (two levels: 10/0 versus 8/0) as a within-subjects factor and age group (five levels) as a between-subjects factor yielded the expected main effect of amount of coins [*F*(1, 88) = 80.09, *p* < 0.001] and a marginally significant main effect of age [*F*(4, 88) = 2.35, *p* = 0.06]. *Post hoc* Tukey comparisons on the age effect revealed that the 20-year-old group offered significantly more than the 8- to 9-year-old group, with the other groups showing intermediate scores.

**Figure 3:**
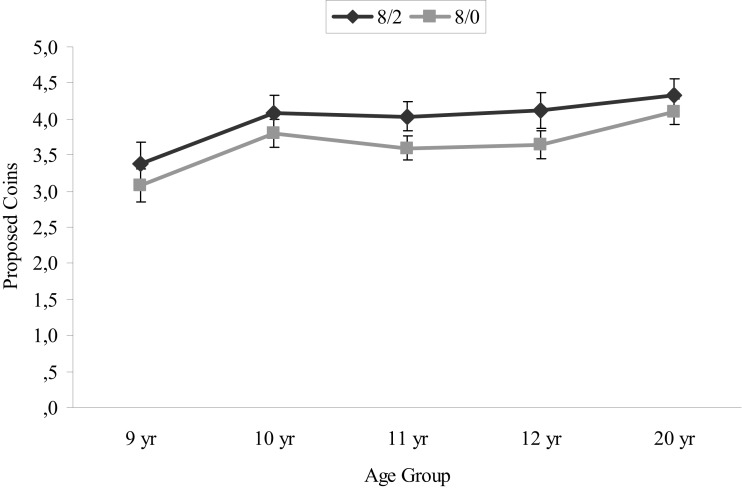
**Mean amount of proposed coins and standard errors in the complete 8/0 condition versus the hidden 8/2 condition in all age groups**.

The second question concerned whether the age groups were differentially sensitive to hidden information *when this increased the total number of coins available*. A repeated measure analysis (ANOVA) with hidden coins (two levels: 8/2 versus 8/0) as a within-subjects factor and age group (five levels) as a between-subjects factor yielded a main effect of hidden coins [*F*(1, 88) = 12.13, *p* < 0.01] and a main effect of age [*F*(4, 88) = 2.99, *p* < 0.05]. There was no hidden coin × age group interaction. The main effect of hidden coins showed that all participants offered more coins when more coins were available in the hidden information condition (8/2) compared to the complete information condition (8/0; see Figure 3). The age effect was examined using Tukey’s b *post hoc* tests and the results showed that 8/9-year-olds kept significantly more coins for themselves by making lower offers in both conditions than the 10-year-olds and the 20-year-olds. Ten and 11-year-olds did not differ significantly from the other age groups.

**Figure 4:**
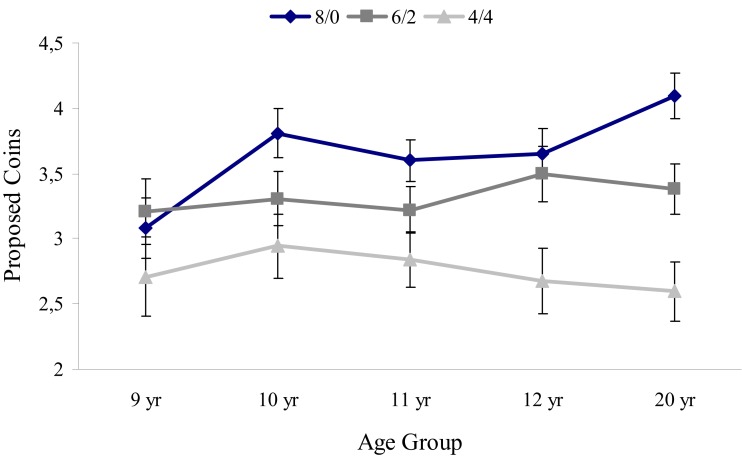
**Mean amount of coins and standard errors offered in the 3 conditions with a total number of 8 coins (i.e., 8/0, 6/2, and 4/4) in five age groups**.

Third, in order to examine age differences in strategic motivations, we compared conditions with varying amounts of hidden coins, *when this decreased the total number of coins available*. A repeated measure analysis (ANOVA) with hidden coins (three levels: 8/0 versus 6/2 versus 4/4) as a within-subjects factor and age group (five levels) as a between-subjects factor again yielded a main effect of hidden number of coins [*F*(1, 88) = 55.32, *p* < 0.01] and a marginally significant interaction between hidden coins and age group [*F*(4, 88) = 2.27, *p* < 0.07; *p*η^2n^ = 0.1, see Figure 4].

To examine whether offers in complete information conditions were significantly different from offers made in the hidden conditions within age groups, a repeated measure (ANOVA) *post hoc* analysis was conducted between the conditions 8/0, 6/2, and 4/4 for each age group separately. The results demonstrated that all age groups differentiate between the three conditions [all *F*s (1, 16–24) > 9.91, all *p*s < 0.01], except for the youngest age group (8/9-year-olds).

The same analysis was performed for the 10 coins condition. Here, the repeated measure analysis (ANOVA) with hidden coins (three levels: 10/0 versus 8/2 versus 6/4) as a within-subjects factor and age group (five levels) as a between-subjects factor again yielded a main effect of hidden number of coins [*F*(1, 88) = 53.16, *p* < 0.001]. Contrary to what we expected, neither a main effect for age (*p* = 0.17) nor an interaction between hidden coins and age group (*p* = 0.66) was significant. Thus, the age differences were restricted to specific conditions, which could indicate that the 8- to 9-year-old group has more variance in responding yielding only some of the age effects significant. In order to determine whether this was the case, we have performed an independent *t*-test to measure the homogeneity between the different age groups for each condition separately. Levene’s test for equality of error variances showed that there was no significant variance between age groups in case of the conditions: 8/0, 8/2, and 6/4. The remaining conditions did show unequal variance between age groups, such that variance was larger in the youngest groups compared to the older age groups: 10/0 (*p* < 0.01), 6/2 (*p* < 0.05), and 4/4 (*p* < 0.01).

Next, we were still interested in testing whether there would be a difference when selecting only the youngest and the oldest age group, based on visual inspection of the graphs. A repeated measure analysis (ANOVA) with hidden coins (three levels: 10/0 versus 8/2 versus 6/4) as a within-subjects factor and age (two levels: 8/9-year-olds versus 20-year-olds) as a between-subjects factor yielded the expected main effect of condition [*F*(1, 31) = 32.11, *p* < 0.01] and the expected main effect of age [*F*(1, 31) = 5.23, *p* < 0.05]. This result is consistent with previous findings reporting lower offers by 8/9-year-olds compared to adults. We note though that this is a *post hoc* analysis and should be interpreted with caution.

### Links with perspective-taking

To examine whether PT changes over time related to the offers made in the UG, correlations were computed between UG conditions and the PT scale of the IRI. The scale did not correlate with coins offered in the complete information conditions (8/0). In contrast, the PT scale was positively related to the number of coins offered in the hidden information condition 8/2 (*r* = 0.21, *p* < 0.05) and related marginally to the hidden information condition 6/4 (*r* = 0.19, *p* = 0.06), and 4/4 (*r* = 0.19, *p* = 0.06). This scale identifies the participants who are able to adopt the perspective of others in real life situations (Davis, 1980). Thus, this association seems to indicate that the hidden information condition required PT.

After controlling for age, PT was positively related to the number of coins offered in all hidden conditions, except for the 8/2 (*r* = 0.23, *p* < 0.05, *N* = 87) condition: 6/2 (*r* = 0.21, *p* < 0.05, *N* = 87), 6/4 (*r* = 0.24, *p* < 0.05, *N* = 87), and 4/4 (*r* = 0.28, *p* < 0.01, *N* = 87). There were no significant correlations with the complete information conditions (8/0 or 10/0).

In addition, we also performed a mediation analysis in order to be able to determine whether PT has a mediating role in the age differences in UG behavior. In the first regression model, age was found to be significantly and positively associated with PT (*b*_1_ = 0.32, *p* < 0.01). In regression model two, the association between PT and amount of coins offered in the 8/2 condition was found to be significantly and positively associated (*b*_2_ = 0.23, *p* < 0.05). The third regression model found that age and amount of offered coins in the 8/2 condition were significant and positively associated (*b*_3_ = 0.21, *p* < 0.05). Finally, when age and PT were included in the same model, the association between age and amount of coins offered in the 8/2 condition was no longer significant (*b*_4_ = 0.3, *p* < 0.08), fulfilling the requirements for full mediation. The Sobel Test confirmed that PT fully mediated the association between age and amount of coins offered (*p* < 0.01). Thus, older participants who scored higher on the self-report PT subscale made higher offers in the specific case of the 8/2 condition. No mediation effects were found for the other conditions.

## Discussion

The goal of this study was to examine the role of strategic versus altruistic motivations in fairness considerations and their developmental trajectories. The results of this study show that by age 8/9 children already show strategic fairness considerations. All participants reduced the number of coins offered when information was hidden, showing that the ability to use strategies already develops at a young age. Specific follow up comparisons revealed age differences in how children and adults used strategies. That is, in standard proposal situations (i.e., with no-hidden information), adults proposed more fair offers than 8/9-year-old children, with children between ages 10 and 12 showing an intermediate pattern. The results further show that by age 8/9 children distinguish less between the hidden and no-hidden information conditions compared to adults who clearly differentiated based on the impending information. It should be noted that it was only in a subset of the conditions that children in the age of 8/9 years-old acted less strategically than adults. Yet, these results may indicate that adults are in certain situations more strategic than children. Below, we interpret these results in the light of our hypotheses.

The first hypothesis that was tested was whether adults show higher levels of concern for others and have altruistic motives for offering fair distributions. The results from the current study suggest the development of more strategic motives during late childhood, given that proposed offers in the complete information conditions increased with age. The average amount of coins adults proposed to the other party in complete information conditions was approximately 50% compared to an average of 40% of the youngest age group. This age-related increase in offers made in complete information conditions is consistent with prior results (Harbaugh et al., 2003; Güroğlu et al., 2009; Steinbeis et al., 2012). Consistent with this point of view, Epley et al. (2004) performed an experiment in which participants had to take the visual perspective of the other person, to see objects from their point of view. The results showed that adults compared to children viewed solutions from different perspectives and were better in controlling their self-centered tendency in taking perspective of others. In this study, strategic motivations can possibly explain the increased prosocial behavior in adults, where they make fair offers to maximize their own outcomes (Reuben and van Winden, 2010). People tend to offer higher amounts when they reason about what other people will find acceptable and what is accepted according to social norms (Straub and Murnighan, 1995). The fear of being rejected by their counterpart prevents proposers to make a low offer (van Dijk and Vermunt, 2000; van Dijk et al., 2004).

The hypothesis of increases in strategic fairness considerations finds support in the analyses of the hidden conditions. The hidden, or incomplete information, conditions allowed for the comparison of offers where information was not available to the responder. All participants were strategic and offered less in the hidden, conditions compared to when all information was available to the responder. At the same time, all participants were also altruistic, because they did not lower their offer to 50% of what was visible to the responder (for example, even in the 4/4 condition, participants offered on average 2.8 coins, which is more than 2 coins which would have been considered a fair split by the responder). A comparison of age groups revealed lower offers by children and adults in case of the hidden 8/2, 6/2, and 4/4 conditions. Adults offered approximately 45% of their amount in the 8/2 condition, and children offered approximately 40%. In case of the 6/2, and 4/4 conditions, however, the offers of children and adults were no longer different from each other. These findings indicate that children already have a basic understanding of different strategic motives, but that behavior of adults is more consistent and seems to be more driven by strategic intentions.

Previous studies already pointed out that adult proposers like to benefit from a favorable situation (Kagel et al., 1995; van Dijk et al., 2004; Koning et al., 2011). The current findings show that children showed a small decrease in number of coins proposed to the other player under hidden conditions, whereas adults dropped their offers to a much less altruistic, self-interested level compared to the complete information condition. Possibly, this reflects more self-oriented decision-making in case of more hidden information in the older age groups. One interpretation of this developmental difference is that the age-related increase in levels of PT, which allows individuals to predict what others find fair or unfair. This hypothesis is supported by our findings that behavior in the hidden conditions was correlated with self-reported PT skills and the outcomes of the mediation analyses revealing that self-reported PT mediates the relation between age and strategic UG behavior.

A second possible mechanism which can explain the developmental difference is the increase in inhibitory control with increasing age. An intriguing study by Steinbeis et al. (2012) revealed a correlation between strategic UG behavior (indexed by the difference score of the UG and the DG) and performance on the stop-signal task, a measure for response inhibition. These differences were associated with developmental changes in the contribution of the dorsolateral prefrontal cortex (DLPFC), a brain region known to develop across childhood and adolescence. Thus, it is possible that besides PT, the ability to control impulsive choices also contributes to the development of strategic bargaining. For example, Steinbeis et al. (2012) described that young children (age 6–9) can already point out when a division is unfair. Yet it is only with increasing age that they act strategically accordingly.

Taken together, this study showed that between ages 8 and 13, children offer more to others in the UG, but also become more strategic in fairness consideration. These findings were interpreted in terms of increasing levels of PT and impulse control. One aspect which received less attention in this study was the role of individual differences. Takagishi et al. (2010) recently reported that half of the children younger than six already reject unequal offers. Thus, there are important individual differences in fairness considerations among children, which can have influenced the robustness of the findings in this study, especially in the younger children. Future studies would therefore greatly benefit from following children’s fairness behavior longitudinally, and relate this development to individual differences in personality, empathy, and prosocial behavior (Eisenberg et al., 2005).

## Conflict of Interest Statement

The authors declare that the research was conducted in the absence of any commercial or financial relationships that could be construed as a potential conflict of interest.
